# Supplementary material for: Dimerization of European Robin Cryptochrome 4a
Source: J Phys Chem B. 2023 Jul 10;127(28):6251–64. doi: 10.1021/acs.jpcb.3c01305 (PMC10364083; doi:10.1021/acs.jpcb.3c01305)
Supplement: Supplementary file 1 — jp3c01305_si_001.pdf [file jp3c01305_si_001.pdf]

Dimerisation of European robin cryptochrome 4a
(Supplementary Material)

Maja Hanić[1#], Lewis M. Antill[2,3#], Angela S. Gehrckens[4#], Jessica Schmidt[5], Katharina Görtemaker[6], Rabea Bartölke[5], Tarick J. El-Baba[4,7], Jingjing Xu[5], Karl-Wilhelm Koch[6,8], Henrik Mouritsen[5,8], Justin L. P. Benesch[4,7], P. J. Hore[4], and Ilia A. Solov'yov[1,8,9*]

[1]*Institute of Physics, Carl von Ossietzky University of Oldenburg, Carl-von-Ossietzky Straße 9-11, 26129, Oldenburg, Germany*

[2]*Graduate School of Science and Engineering, Saitama University, 255 Shimo-okubo, Sakura Ward, Saitama 338-8570, Japan*

[3]*JST, PRESTO, 4-1-8 Honcho, Kawaguchi, Saitama 332-0012, Japan*

[4]*Department of Chemistry, University of Oxford, Physical & Theoretical Chemistry Laboratory, South Parks Road, Oxford OX1 3QZ, UK*

[5]*Department of Biology and Environmental Sciences, Carl von Ossietzky University of Oldenburg, Carl-von-Ossietzky Straße 9-11, 26129, Oldenburg, Germany*

[6]*Department of Neuroscience, Division of Biochemistry, Carl von Ossietzky University of Oldenburg, D-26111, Oldenburg, Germany*

[7]*Kavli Institute for NanoScience Discovery, Dorothy Crowfoot Hodgkin Building, University of Oxford, OX1 3QU*

[8]*Research Center for Neurosensory Sciences, Carl von Ossietzky University of Oldenburg, Carl-von-Ossietzky Straße 9-11, 26111, Oldenburg, Germany*

[9]*Center for Nanoscale Dynamics (CENAD), Carl von Ossietzky Universität Oldenburg, Ammerländer Heerstr. 114-118, 26129 Oldenburg, Germany*

*ilia.solovyov@uni-oldenburg.de

#These authors contributed equally

Table of Contents

Experimental supplementary figures 2

Full SDS-PAGE gel, peak isolation using tandem native MS and XL-MS results 2

Full SDS-PAGE gels displaying the degree of covalent dimerisation over time 3

Photometric cysteine exposure measurements 4

Experimental supplementary tables 5

Computational supplementary material 7

*Er*Cry4a non-covalent dimers 8

*Er*Cry4a 317 dimer family 11

*Er*Cry4a cov^D – a dimer with two disulphide bonds 13

*Er*Cry4a 189 dimer family 15

*Er*Cry4a 412 dimer family 17

Distance analysis between cysteine residues 22

Molecular dynamics simulation protocol 30

Involvement of the C-terminal in dimer formation 32

Comparison of cross-link experiment with MD simulation data 36

References 37

Experimental supplementary figures

Full SDS-PAGE gel, peak isolation using tandem native MS and XL-MS results

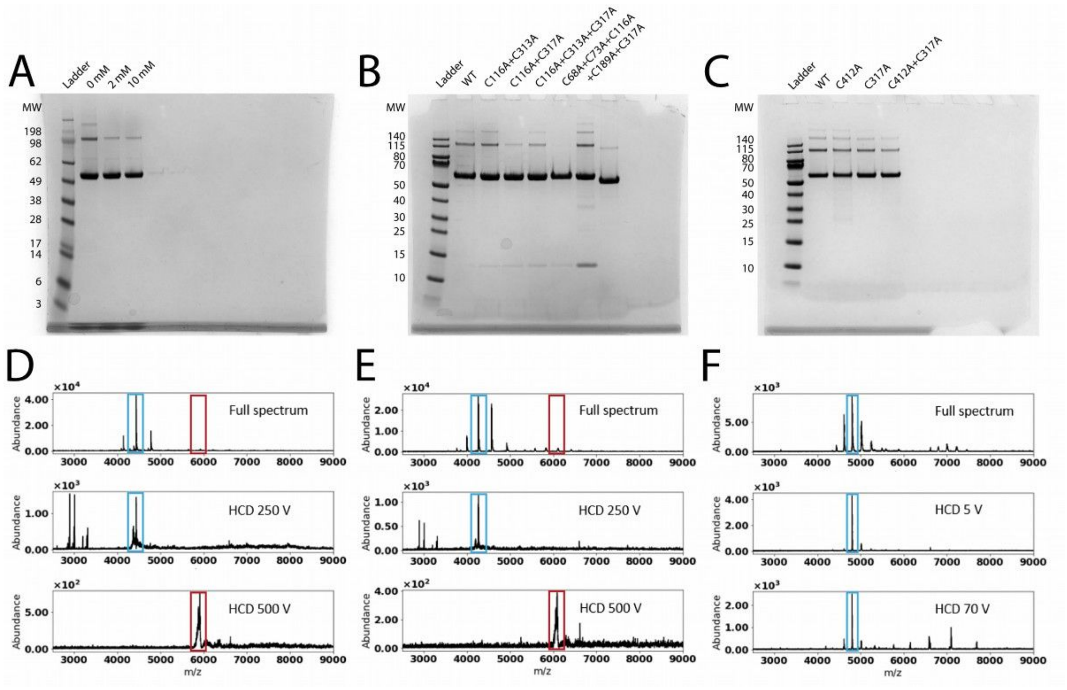

Figure S1: (**A**)-(**C**) Denaturing SDS-PAGE gels run for the different *Er*Cry4a proteins displayed in the native MS spectra shown in the main text. Proteins are as indicated at the tops of the columns. All leftmost columns display protein ladders. In (**A**) the SeeBlue^{TM} Plus2 Pre-stained Protein Standard ladder (Invitrogen) was used and in (**B**) and (**C**) the PageRuler Prestained Protein Ladder (Thermo Scientific) was used with the molecular weight (MW) mass markers in kDa as indicated. The columns displayed with no indicator are not relevant for the present investigation. (**D**)-(**F**) Native MS in combination with tandem MS was used to isolate a monomer and a dimer peak of *Er*Cry4a WT (**D**) (without its His-tag) and *Er*Cry4a C317S (**E**) (with His-tag; shipped in 10 mM BME to prevent higher order oligomerisation during transport) to compare their stability upon exposure to high HCD (high energy collisional dissociation) energies. The first rows of both (**D**) and (**E**) show the full spectra, the second the isolated dimer peaks and the third the isolated monomer peaks. HCD values were applied as indicated in the spectra. The spectra displayed for comparison in (**F**) were of CRP (C-reactive protein), a pentameric protein known to be non-covalently bound[1]. The first row shows the full spectrum, the second an isolated pentamer peak and the third shows the isolated pentamer peak after applying an HCD value of 70 V, displaying how it falls apart into smaller subunits at comparatively low HCD energies. The *Er*Cry4a dimers still did not dissociate when the highest HCD energies possible on the instrumental setup (500 V) were applied. The coloured rectangles show which peaks were isolated using tandem MS.

1

Full SDS-PAGE gels displaying the degree of covalent dimerisation over time

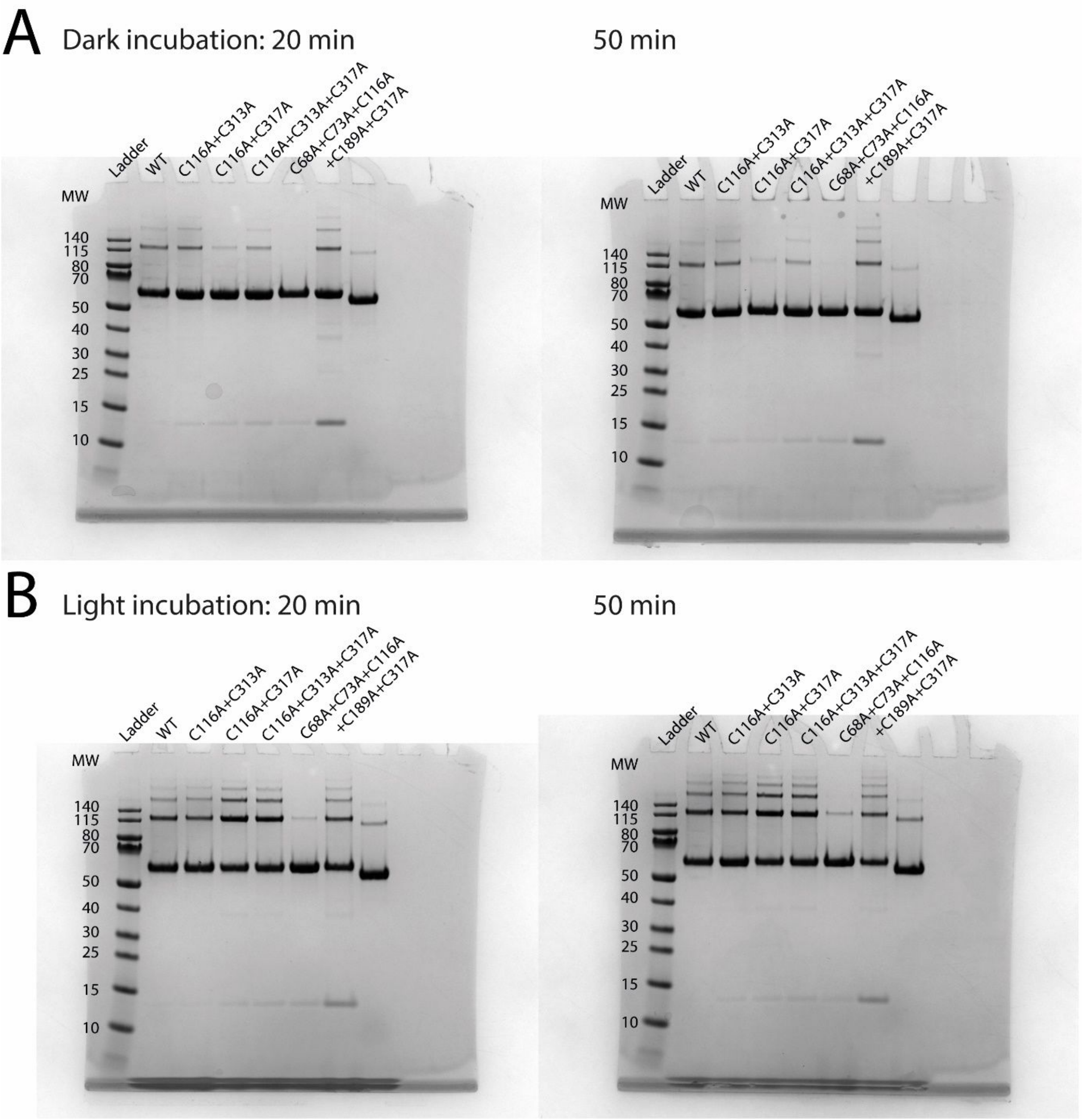

Figure S2: Denaturing SDS-PAGE gels, parts of which are shown in the main text. Proteins are as indicated at the tops of the columns. Both of the leftmost columns display the PageRuler Prestained Protein Ladder (Thermo Scientific) with the molecular weight (MW) mass markers in kDa as indicated. The samples in (A) were kept in darkness and the samples in (B) were incubated under blue light. The columns displayed without labels are not relevant for the present investigation. The SDS-PAGE gel on the left side of (A) is the same as displayed in Fig. S1 (B) and is repeated here for easier comparison.

Photometric cysteine exposure measurements

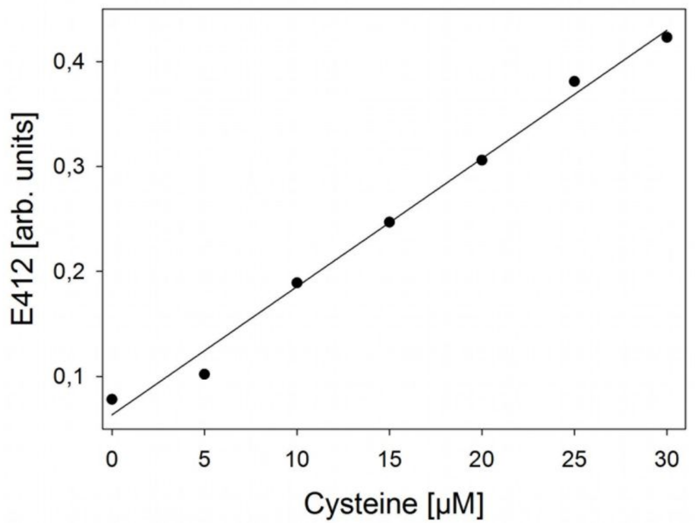

Figure S3: Cysteine accessibility calibration curve. 60 µM of DTNB were added to 0, 5, 10, 15, 20, 25 and 30 µM of L-cysteine. After 10 min incubation at room temperature the absorbance was measured at 412 nm. The *y*-axis shows the absorbance of TNB at 412 nm resulting from the reaction of DTNB with the thiol groups of the cysteines. The solid line comes from a linear regression analysis and has slope 0.0124 μM^{-1}.

2

Experimental supplementary tables

Table S1: Absorbance of 5 µM *Er*Cry4a wildtype (WT) and its mutants measured at 412 nm after 10 min incubation with 60 µM DTNB and the corresponding number of accessible cysteines.

WT absorption	Accessible cysteines	C317A absorption	Accessible cysteines	C412A absorption	Accessible cysteines	C116-C317 absorption	Accessible cysteines
0.30	4.77	0.26	4.13	0.27	4.17	0.16	3.12
0.30	4.76	0.26	4.09	0.27	4.14	0.17	3.30
0.28	4.58	0.28	4.34	0.27	4.14	0.18	3.35
0.30	4.82	0.28	4.30	0.27	4.25	0.17	3.30
0.31	4.98	0.28	4.31	0.26	4.09	0.18	3.43

Table S2: XL-MS results. Only disulphide bonds and crosslinks between peptides containing lysine residues[2] that were identified with a MeroX score greater than 50 were considered candidates for further analysis. The residues in brackets clarify the position of the linked residue within each peptide.

Link	Experiment	Score	Peptide 1	Peptide 2	Linked residues
DSBU	1 (dimer)	81	144-158 (K14)	144-158 (K14)	K152-K152
		76	224-241 (K16)	434-440 (K1)	K234-K429
DSBU	1 (monomer)	59	224-241 (K16)	434-440 (K1)	K234-K429
DSSO	1 (dimer)	52	224-241 (K16)	516-526 (K9)	K234-K519
DSSO	1 (monomer)	109	144-158 (Y13)	503-514 (K10)	K152-K507
disulphide	1 (dimer)	144, 106, 90	415-420 (C3)	415-420 (C3)	C412-C412*
		103, 97	362-370 (C5)	415-420 (C3)	C361-C412
		120	460-467 (C4)	415-420 (C3)	C458-C412
disulphide	1 (monomer)	121, 97, 90, 79	415-420 (C3)	415-420 (C3)	C412-C412*
		117	460-467 (C4)	415-420 (C3)	C458-C412
disulphide	2 (monomer)	145, 101, 97, 94, 93	362-370 (C5)	415-420 (C3)	C361-C412
		164, 149, 81	415-420 (C3)	415-420 (C3)	C412-C412
		166, 109	460-467 (C4)	415-420 (C3)	C458-C412
		95	362-370 (C5)	460-467 (C4)	C361-C458

*A disulphide bond between two C412 residues was found in both the monomer and dimer fractions, possibly due to cross-contamination between the two fractions on the SDS-PAGE gel.

Computational supplementary material

Table S3: Solvent exposure and locations of the cysteine residues in WT *Er*Cry4a calculated as described in the Methods section and Eq. (1) in the main text. The values were averaged over the duration of the production MD simulation.

Cysteine	Secondary structure	Solvent exposure / %
317	unspecified turn	71.9
116	α-helix	57.5
189	α-helix	55.5
68	α-helix	44.7
412	coil, close to α-helix	30.6
73	coil, close to β-strand	22.5
179	coil, close to α-helix	6.5
313	unspecified turn	6.1
458	coil	1.7
361	α-helix	1.1
257	unspecified turn	0.2

*Er*Cry4a non-covalent dimers

The full length *Er*Cry4a WT structure (1-527) was used to produce the stable non-covalently bound dimer $ncov^A$, and a truncated sequence (8-495) was used to construct the $ncov^M$ dimer (illustrated in Fig. S4Figure S4). Several analyses were performed on the simulated dimers: the results are summarised in Table S4 and Fig.Figure S5. The average $\overline{RMSD}$ values calculated over the duration of the production simulation are shown in Table S4, while the time evolution of the $\overline{RMSD}$ is presented in Fig. Figure S5. The $ncov^A$ and $ncov^M$ dimers appear to be rather stable with an average $\overline{RMSD}$ value of 3.51 ± 0.36 Å and 3.36 ± 1.36 Å, respectively, whereas $ncov^4$ is rather unstable with an average $\overline{RMSD}$ value of 8.98 ± 2.00 Å. The contrast in the stabilities of the three dimers is attributed to the differences in interaction energy and the hydrogen bonding network, both factors being much stronger in $ncov^A$ and $ncov^M$ (see Table S4).

Average interaction energies, E_{tot}, for the non-covalent dimer family are given in Table S4. The $ncov^A$ dimer was selected for further comparative analysis because of its exceptionally strong interaction energy that already manifests itself after the 2 ns equilibration simulation (E_{tot} = −928 ± 25 kcal mol^{-1}, Table S5). The $ncov^4$ dimer was considered interesting because of its similar spatial arrangement to the covalent dimer cov^{317A} discussed below. Considering that the full length *Er*Cry4a protein was used for $ncov^4$ and $ncov^A$ simulations, the presence of the CTT might have added to the stability of the dimer by contributing favourably to the resulting interaction energies.

The average value of the radius of gyration, R_g, was computed for the non-covalent dimers, where the averaging was over the span of the MD trajectories. The results in Table S4 reveal that R_g for $ncov^A$ and $ncov^M$ is significantly lower than for $ncov^4$, which indicates that the protein structures are more compact.

The average $\overline{RMSF}$ value turns out to be smallest for $ncov^A$ (Table S4), where the largest fluctuations occur in the CTT domain (residues 498-527) as one would expect for an intrinsically disordered region of the protein (see Fig. Figure S5D-F).

There are 106 and 111 inter-monomer hydrogen bonds in the $ncov^A$ and $ncov^M$ dimers, respectively, while only 40 exist in $ncov^4$. The numbers of inter-monomer salt bridges in $ncov^4$ and $ncov^A$ are similar and significantly larger than in $ncov^M$.

The interaction surface areas of $ncov^A$ and $ncov^M$ are more than double that of $ncov^4$. Interestingly, even without the CTT, $ncov^M$ has a bigger interaction surface than $ncov^4$. Taking all the factors into consideration, $ncov^A$ and $ncov^M$ were selected as the two most promising non-covalent *Er*Cry4a candidates for a further comparative analysis.

Table S4: Summary of the characteristics of the non-covalent dimers. The table includes the computed values of the radius of gyration R_g, the average ($\overline{RMSD}$) and ($\overline{RMSF}$) values, the total area of the binding interface A_{IS}, and the total number of inter-monomer hydrogen bonds and salt bridges. Only salt bridges present in more than 10% of the MD frames were counted. All values have been averaged over the duration of the corresponding MD simulations.

Dimer	$-E_{tot}$ / kcal mol^{-1}	R_g / Å	$\overline{RMSD}$/Å	$\overline{RMSF}$/Å	A_{IS} / Å^2	Hydrogen bonds	Salt bridges
$ncov^4$	426±95	41.8±0.9	9.0±2.0	3.2±1.5	1125±161	40	33
$ncov^A$	857±85	35.2±0.3	3.5±0.4	2.2±1.0	2762±292	106	29
$ncov^M$	505±163	33.0±0.3	3.4±1.4	2.3±1.0	2442±284	111	12

Table S5: Ten most favourable non-covalently bound full length *Er*Cry4a dimers, $ncov^n$, produced by ZDOCK. Average interaction energies E_{tot} between each monomeric subunit after 2 ns equilibration are shown together with the van der Waals, E_{vdw}, and Coulomb, E_{elec}, contributions.

Dimer	$-E_{elec}$ / kcal mol^{-1}	$-E_{vdW}$ / kcal mol^{-1}	$-E_{tot}$ / kcal mol^{-1}
$ncov^1$	320±61	91±8	411±65
$ncov^2$	237±47	114±12	351±42
$ncov^3$	332±41	118±13	450±36
$ncov^4$	245±37	73±13	318±33
$ncov^5$	106±44	65±8	171±41
$ncov^6$	513±41	111±10	624±42
$ncov^7$	245±42	102±8	347±43
$ncov^8$	283±29	55±7	338±27
$ncov^9 = ncov^A$	826±66	102±8	928±25
$ncov^{10}$	319±24	51±4	370±25

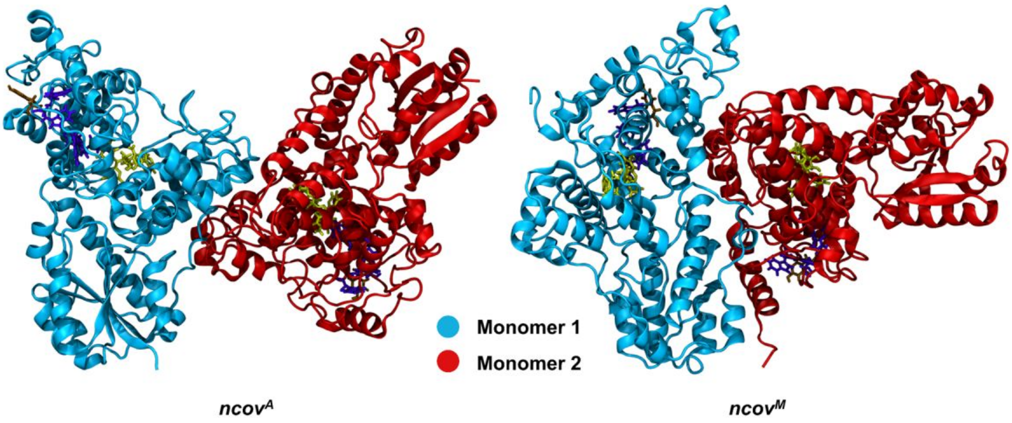

Figure S4: Structures of the non-covalently bound dimers ncov^{A} and ncov^{M}. The FAD cofactor, Trp-tetrad (W395, W372, W318, W369), and Y319 are shown in yellow, violet and ochre, respectively.

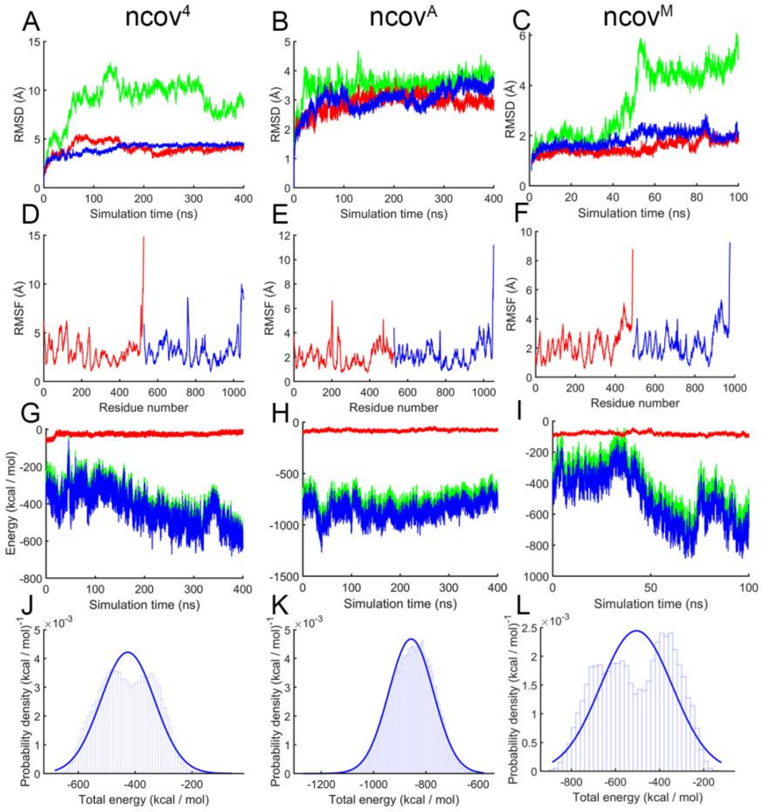

Figure S5: Time-evolution of the root mean square displacement (RMSD) values computed for the backbone atoms of the non-covalently bound dimers ncov^{4}, ncov^{A} and ncov^{M} during the 400 ns production simulation (A, B, C). Monomers (blue and red); dimer (green line). Average root mean square fluctuations $\overline{\boldsymbol{RMSF}}$ computed for the residues of the non-covalently bound dimers ncov^{4}, ncov^{A} and ncov^{M} (D, E, F). Red corresponds to monomer A and blue to monomer B. (G, H, I) Time-evolution of the interaction energies for non-covalently bound dimers ncov^{4}, ncov^{A} and ncov^{M}. Red and green denote respectively the van der Waals and Coulomb contributions to the total interaction energy between the monomers, shown in blue. (J, K, L) Probability density distributions of the interaction energies.

*Er*Cry4a 317 dimer family

Residue C317 is of interest in the context of covalent dimerisation of *Er*Cry4a as it is close to the Trp tetrad (which is involved in magnetic sensing) and is the most solvent-exposed cysteine residue in WT *Er*Cry4a (Fig. 4A and Table S3). C317 is also considered a promising linking residue, as a result of the experiments described in the main text. For this and all other covalent dimers, a truncated sequence (8-495) was used to construct the dimers. This truncation was based on the sequence used for the structure determination of pigeon Cry4a[3].

Three different dimeric structures were constructed to investigate the involvement of C317 in covalently-bound *Er*Cry4a dimers (see Table S16). Dimers cov^{317A} and cov^{317B} are illustrated in Fig. **Figure S6**. Table S6 gives the average $\overline{RMSD}$ values for the three dimers covalently linked through the C317 residue. The time-dependence of the RMSD is given in Fig. Figure **S7**A-C. The results in Table S6 demonstrate that cov^{317A} and cov^{317B} are much more stable than cov(317)[3]. Table S6 also gives the average $\overline{RMSF}$ values which provide information on the flexibility of the three structures. The average $\overline{RMSF}$ is smallest for cov^{317A}, indicating that the structure is least flexible, and unusually large for cov^{317B}.

The analysis of the average radius of gyration, R_g, in Table S6 shows that R_g is comparable for all three structures indicating that their geometric shapes are similar, which is also suggested by their similar interaction surface areas. Furthermore, the interaction energy of the subunits shown in Fig. Figure **S7**D-F, shows that the most favourable interactions occur between the subunits of the cov^{317A} dimer. The hydrogen bonding network is more elaborate for the cov(317)[3] dimer; here the number of hydrogen bonds could be directly related to the larger interaction surface area. The salt bridge analysis favours cov^{317A}, supporting this dimer as the representative candidate of the *Er*Cry4a C317 dimer family.

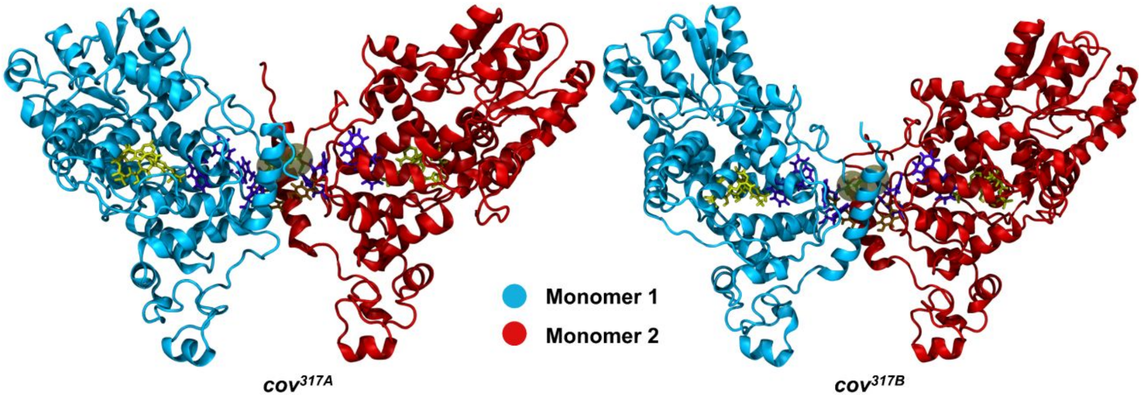

Figure S6: Structures of the covalently bound dimers of *Er*Cry4a, cov^{317A} and cov^{317B}. The FAD cofactor, Trp-tetrad (W395, W372, W318, W369), and Y319 are shown in yellow, violet and ochre, respectively. Brown spheres indicate the approximate position of the C317-C317 disulphide bond between the monomers.

Table S6: Summary of the characteristics of the cov(317)^n dimer family. The table includes the computed values of the radius of gyration R_g, the average $\overline{RMSD}$ and $\overline{RMSF}$ values, the total area of the binding interface A_{IS}, as well as the total number of inter-monomer hydrogen bonds and salt bridges. Only salt bridges present in more than 10% of the MD frames were counted. All values have been averaged over the duration of the corresponding MD simulations.

Dimer	$-E_{tot}$ / kcal mol^{-1}	R_g / Å	$\overline{RMSD}$ / Å	$\overline{RMSF}$ / Å	A_{IS} / Å^2	Hydrogen bonds	Salt bridges
cov^{317A}	186±103	38.3±0.3	4.0±0.6	1.8±0.9	1082±220	64	10
cov^{317B}	171±73	38.5±0.2	3.8±0.8	1.8±0.9	1070±110	59	12
cov(317)^3	–42±96	35.7±1.3	8.2±2.9	10.9±2.9	1173±198	83	6

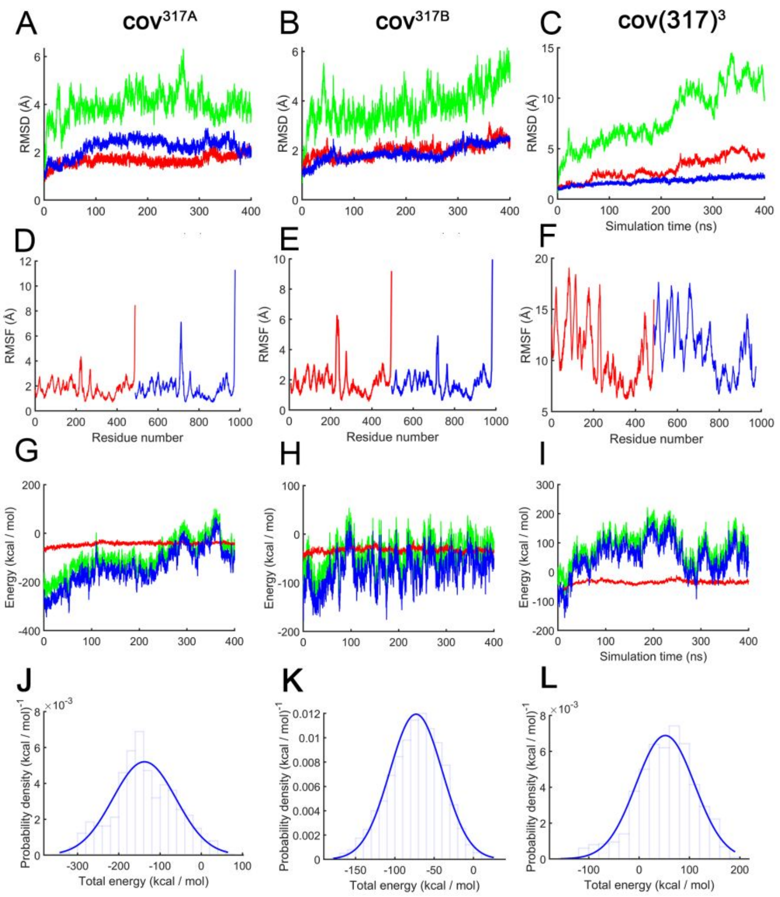

Figure S7: Time evolution plots for the *Er*Cry4a C317 dimer family. (**A-C**): Root mean square deviation (RMSD) of the backbone atoms of three members of the *Er*Cry4a C317 dimer family, computed for the dimers (green) and monomers (red and blue), after equilibration. (**D-F**): RMSF values shown per residue. Residues were represented by C_α atoms. (**G-I**): Time evolution of the interaction energies between two monomeric subunits of the *Er*Cry4a dimers. Red and green denote respectively the van der Waals and Coulomb contributions to the total interaction energy between the monomers, shown in blue. (**J-L**): Probability density distributions of the interaction energies.

*Er*Cry4a cov^D – a dimer with two disulphide bonds

Figure S1 shows that *Er*Cry4a dimers can still be formed after mutation of important cysteine residues that account for a part of the dimerisation. Figure S1 also indicates that small amounts of higher order oligomers exist in addition to the more prominent monomers and dimers, suggesting that several oligomerisation surfaces in *Er*Cry4a may exist and therefore that more than one disulphide bridge could be involved in linking monomers. Oligomers were also found for *Arabidopsis thaliana* cryptochrome 2 (*At*Cry2) where a tetrameric structure is important in the regulation of plant growth[4,5]. Another experimental indication of dimer stability involves the use of higher HCD which demonstrates that *Er*Cry4a dimers do not disintegrate easily (see Figure S1). This result suggests that a covalent linkage between the subunits might involve more than just one disulphide bond. Using M-ZDOCK[6], a tool that symmetrically docks multimers, a dimeric structure was found in which C116 in each of the monomers was close to C313 in the other monomer (see Table S19). This led the construction of a potential stable *Er*Cry4a dimer containing two covalent bonds between the monomers, cov^D = cov(116^{A}313^B - 313^{A}116^B), where A and B stand for monomers (Figure S8).

Table S7 shows the $\overline{RMSD}$ values indicating that the dimeric cov^D structure is stable. A full temporal analysis of *RMSD* and *RMSF* is shown in Figure S9. Furthermore, the $\overline{RMSF}$ analysis indicates low flexibility, while the interaction energy between the two subunits, averages at -178 ± 44 kcal mol^{-1} and is comparable with the values for cov^{317A} and cov^{317B}, even though the interaction surface area for cov^D is twice those of the cov(317) dimers. Figure S8 shows that the *Er*Cry4a cov^D dimer has an inversion centre (centre of symmetry) which is not found for any of the dimeric structures presented in Fig. 1 in the main text.

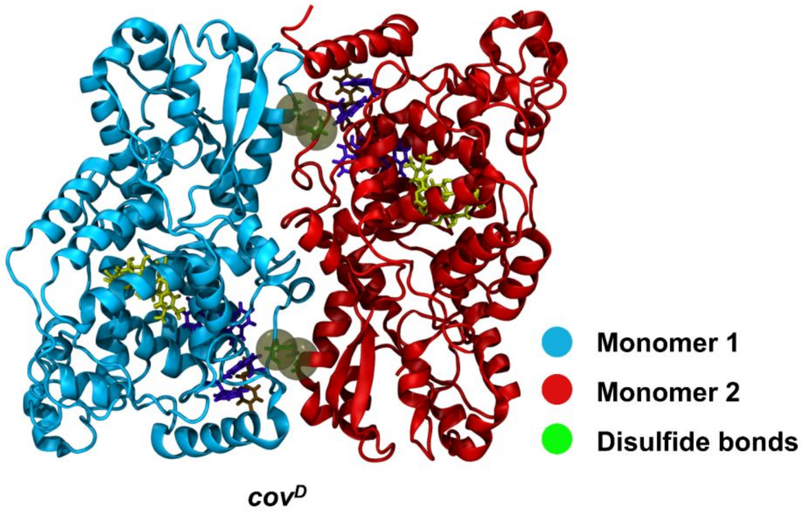

Figure S8: Structure of the doubly covalently bound *Er*Cry4a cov^D dimer. The FAD cofactor, Trp-tetrad (W395, W372, W318, W369), and Y319 are shown in yellow, violet and ochre, respectively. Brown spheres indicate the approximate position of the C116-C313 disulphide bonds between the monomers.

Table S7: Summary of the analysis of the cov^D dimer similar to the results shown in Tables S4 and S6. Interaction energy values shown here account only for non-bonded interaction between the two monomeric subunits. Only those hydrogen bonds appearing between two subunits are counted. A_{IS} is the interaction surface area between the two monomeric subunits.

Dimer	$-E_{tot}$ / kcal mol^{-1}	R_g / Å	$\overline{RMSD}$ / Å	$\overline{RMSF}$ / Å	A_{IS} / Å^2	Hydrogen bonds	Salt bridges
cov^D	178 ± 44	33.0 ± 0.2	2.5 ± 0.3	1.3 ± 0.7	2140 ± 330	47	3

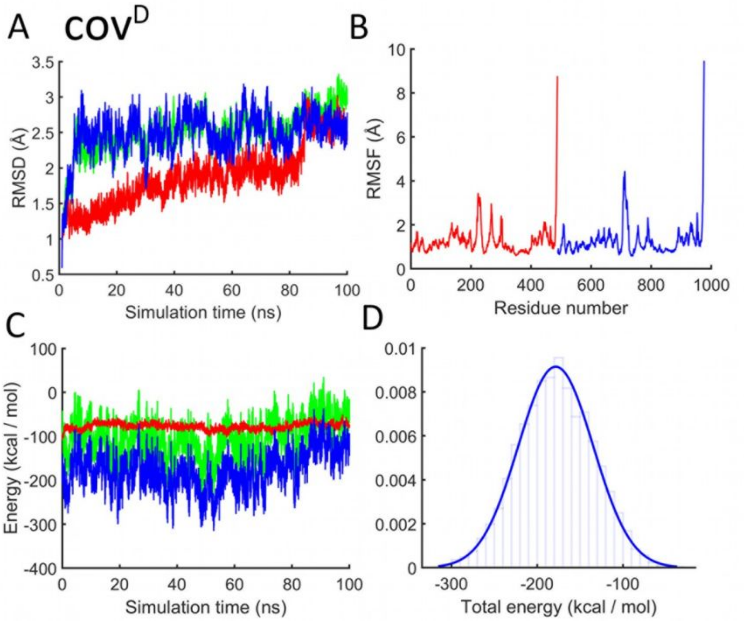

Figure S9: Time evolution of the root mean square deviation (RMSD) of the backbone atoms of the *Er*Cry4a cov^D dimer, computed relative to the structure of the corresponding dimer after equilibration (**A**). Average root mean square fluctuations $\overline{RMSF}$ values computed for the residues of the cov^D dimer (**B**). Red corresponds to monomer A and blue to monomer B. (**C-D**): Time evolution of the interaction energies between two monomeric subunits of the cov^D *Er*Cry4a dimer and the corresponding probability density distribution of the interaction energy. Red and green denote respectively the van der Waals and Coulomb contributions to the total interaction energy between the monomers, shown in blue.

*Er*Cry4a 189 dimer family

Cys189, the third most solvent-exposed residue of monomeric *Er*Cry4a, was found to have a possible binding motif to the Cys458 residue from another monomer, suggesting a dimer with a disulphide bond between the Cys189 and Cys458, denoted cov^{189A} (see Table S15).

Computational analysis reveals that cov^{189A} is stable, with a low RMSF. Although its interaction surface is not particularly large compared to some of the other covalent dimers, the interaction energy of its monomers, –533 kcal mol^{-1}, is the largest of all the covalent dimers studied. Figure S10 shows the spatial orientation of the monomeric subunits; this structure does not have inversion symmetry. Figure S11 shows the temporal analysis of the RMSD, RMSF and E_{tot}.

Table S8: Summary of the analysis of the cov^{189A} dimer. E_{tot} values account only for the non-bonded interaction between the two monomeric subunits. Only those hydrogen bonds that link the subunits are counted.

Dimer	$-E_{\text{tot}}$ / kcal mol^{-1}	R_g / Å	$\overline{RMSD}$ / Å	$\overline{RMSF}$ / Å	A_{IS} / Å^2	Hydrogen bonds	Salt bridges
cov^{189A}	533 ± 122	32.8 ± 0.3	2.2 ± 0.5	1.4 ± 0.5	1407 ± 189	45	11

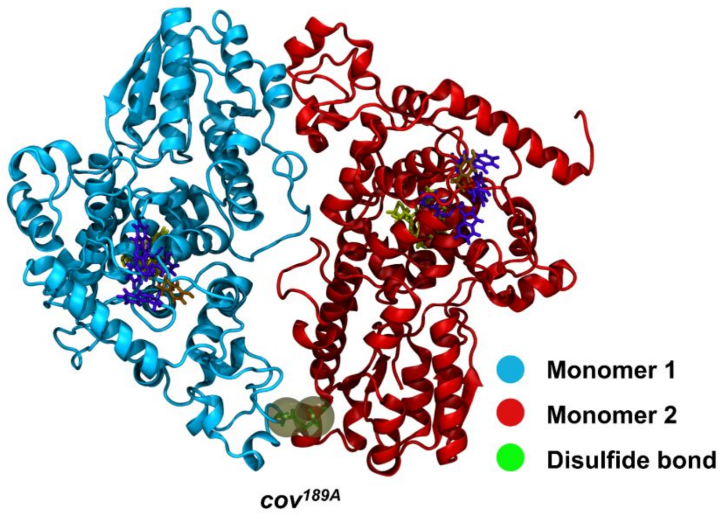

Figure S10: Structure of the *Er*Cry4a cov^{189A} dimer. The FAD cofactor, Trp-tetrad (W395, W372, W318, W369), and Y319 are shown in yellow, violet and ochre, respectively. Brown spheres indicate the approximate position of the C189-C458 disulphide bond between the monomers.

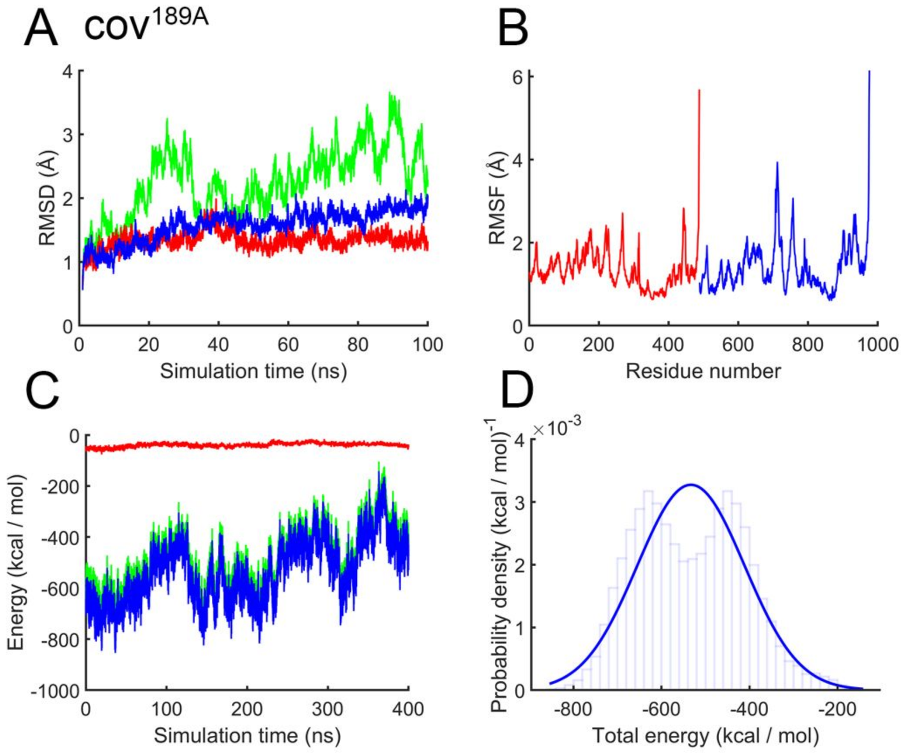

Figure S11: Time evolution of the root mean square deviation (RMSD) of the backbone atoms of the *Er*Cry4a cov^{189A} dimer, computed relative to the structure of the corresponding dimer after equilibration (**A**). Average root mean square fluctuations $\overline{RMSF}$ values computed for the residues of the cov^{189A} dimer (**B**). Red corresponds to monomer A and blue to monomer B. (**C**-**D**): Time evolution of the interaction energies between two monomeric subunits of the cov^{189A} *Er*Cry4a dimer and the corresponding probability density distribution of the interaction energy. Red and green denote respectively the van der Waals and Coulomb contributions to the total interaction energy between the monomers, shown in blue.

*Er*Cry4a 412 dimer family

Residue C412 was also investigated computationally as a possible linker of covalent *Er*Cry4a dimers (Table S17). Eight structures were created: cov(412)$^{1,2,3,A,B,6\text{-}8}$.

The structures of the most stable dimers, cov^{412A} and cov^{412B} (Figure S12), display a symmetric orientation of monomeric subunits relative to one another. Table S9 shows their $\overline{RMSD}$ values: cov^{412A} and cov(412)^{7} appear to be the most stable. cov^{412A} has the smallest $\overline{RMSF}$ (see Figure S14). The interaction energies (Table S9 and **Figure S15**) indicate that cov^{412A} and cov^{412B} should be the most stable of the dimers, even though cov^{412B} would be considered highly unstable based on the $\overline{RMSD}$ analysis (see Figure S13). The strongest interaction energy (for cov^{412A}) is accompanied by the largest interaction surface area, suggesting that cov^{412A} is the most stable dimer from the 412 family.

Table S9: Summary of the analysis of the cov(412)^{n} dimer family similar to the results shown in Tables S4, S6, S7, S8. Interaction energy values shown here account only for non-bonded interaction between the two monomeric subunits. Only those hydrogen bonds appearing between two subunits are counted. A_{IS} is the interaction surface area between the two monomeric subunits.

Dimer	$-E_{tot}$ / kcal mol^{-1}	R_g / Å	$\overline{RMSD}$ / Å	$\overline{RMSF}$ / Å	A_{IS} / Å^2	Hydrogen bonds	Salt bridges
cov(412)^{1}	−315 ± 86	36.9 ± 0.3	3.5 ± 1.0	6.0 ± 1.1	1570 ±257	64	1
cov(412)^{2}	−207 ± 97	38.9 ± 0.5	6.7 ± 2.3	7.8 ± 1.9	1201 ± 280	66	5
cov(412)^{3}	386 ± 111	41.3 ± 0.4	3.9 ± 1.3	5.6 ± 1.2	1069 ± 330	40	20
cov(412)^{4}= cov^{412A}	526 ± 83	41.2 ± 0.2	2.5 ± 0.4	5.7 ± 1.3	1840 ± 115	31	13
cov(412)^{5}= cov^{412B}	437 ± 127	41.4 ± 0.7	6.2 ± 2.8	5.4 ± 1.5	1040±310	49	12
cov(412)^{6}	−87 ± 85	36.8 ± 0.4	3.1 ± 1.0	6.8 ± 1.2	1689 ± 248	65	5
cov(412)^{7}	−145 ± 53	38.1 ± 0.5	1.9 ± 0.4	7.0 ± 1.0	1274 ± 146	29	7
cov(412)^{8}	−382 ± 97	37.2 ± 0.4	2.9 ± 0.9	4.8 ± 0.7	1881 ± 371	75	4

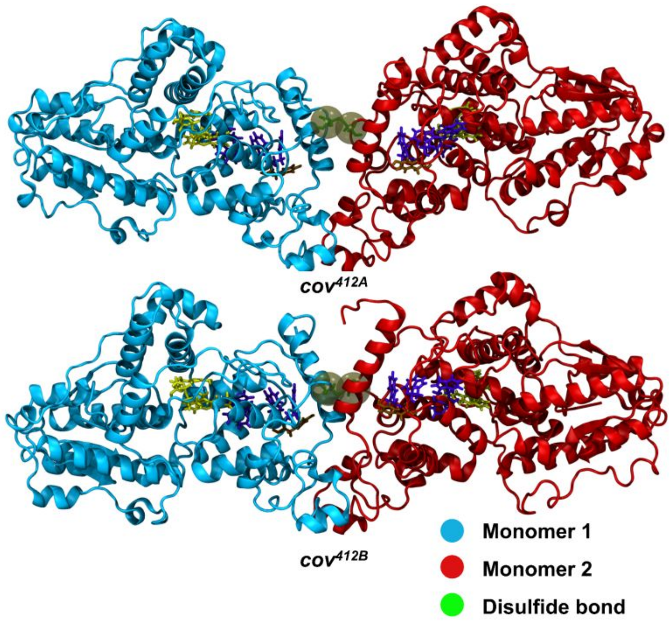

Figure S12: Structures of the two favourable *Er*Cry4a dimers cov^{412A} and cov^{412B}. The FAD cofactor, Trp-tetrad (W395, W372, W318, W369), and Y319 are shown in yellow, violet and ochre, respectively. Brown spheres indicate the approximate position of the C412-C412 disulphide bond between the monomers.

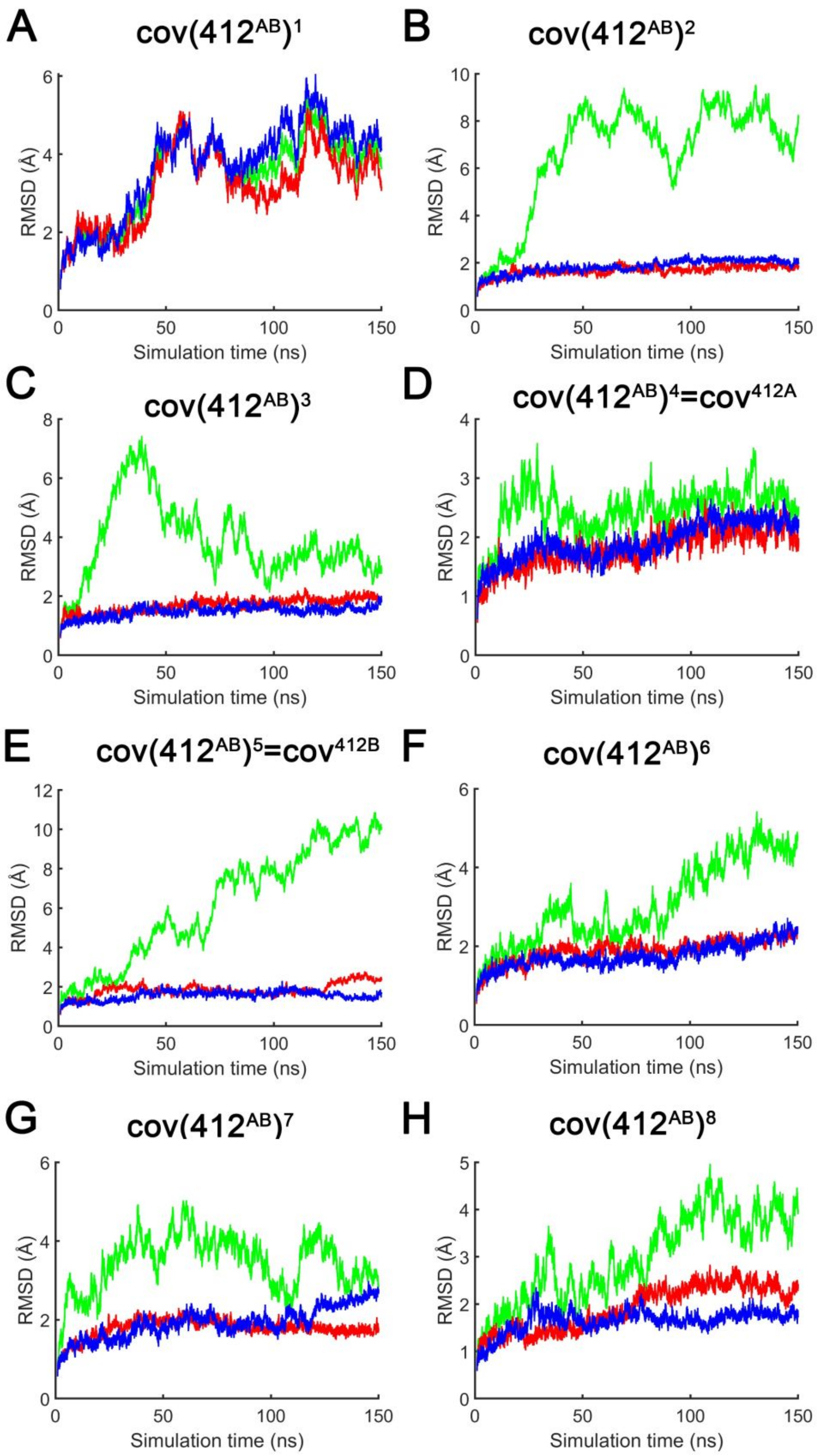

Figure S13: Time-evolution of the RMSD values for the *Er*Cry4a dimeric structures of the cov(412)^n family, computed for each monomeric subunit (blue and red), and the whole dimer (green) relatively to the structures obtained after equilibration simulations.

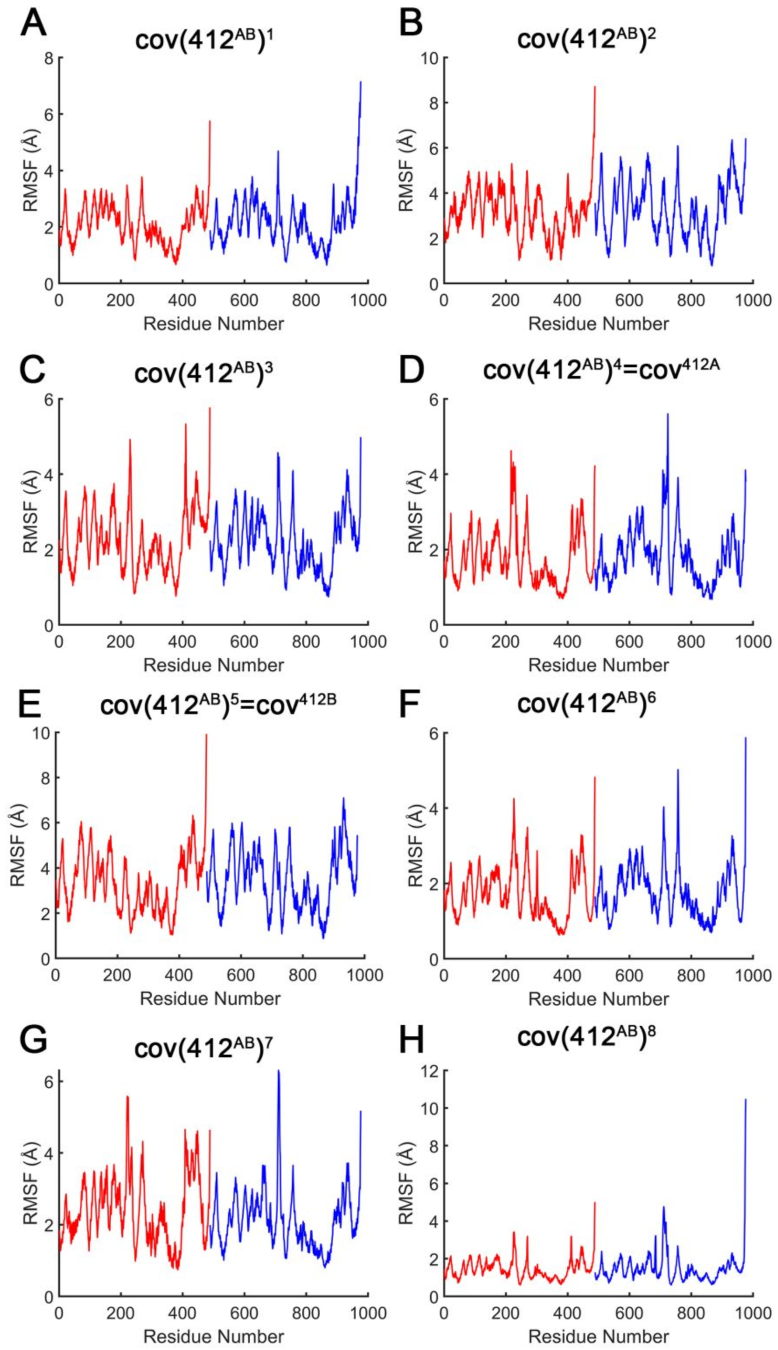

Figure S14: Average root mean square fluctuations $\overline{RMSF}$ values computed for the residues of the *Er*Cry4a dimeric structures of the cov(412)^{n} family. Red corresponds to monomer A and blue to monomer B.

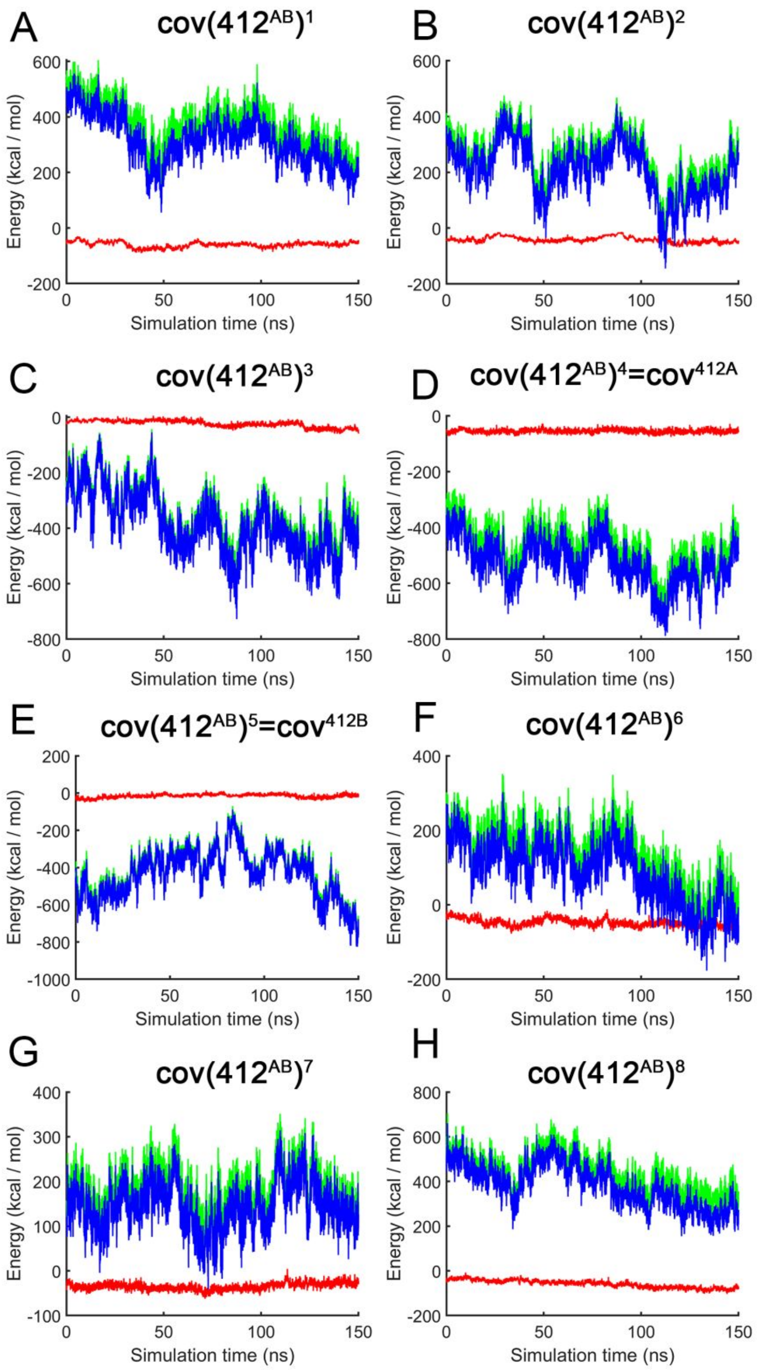

Figure S15: Time evolution of the interaction energies between two monomeric subunits of the cov(412)^{n} *Er*Cry4a dimers. Red and green denote respectively the van der Waals and Coulomb contributions to the total interaction energy between the monomers, shown in blue.

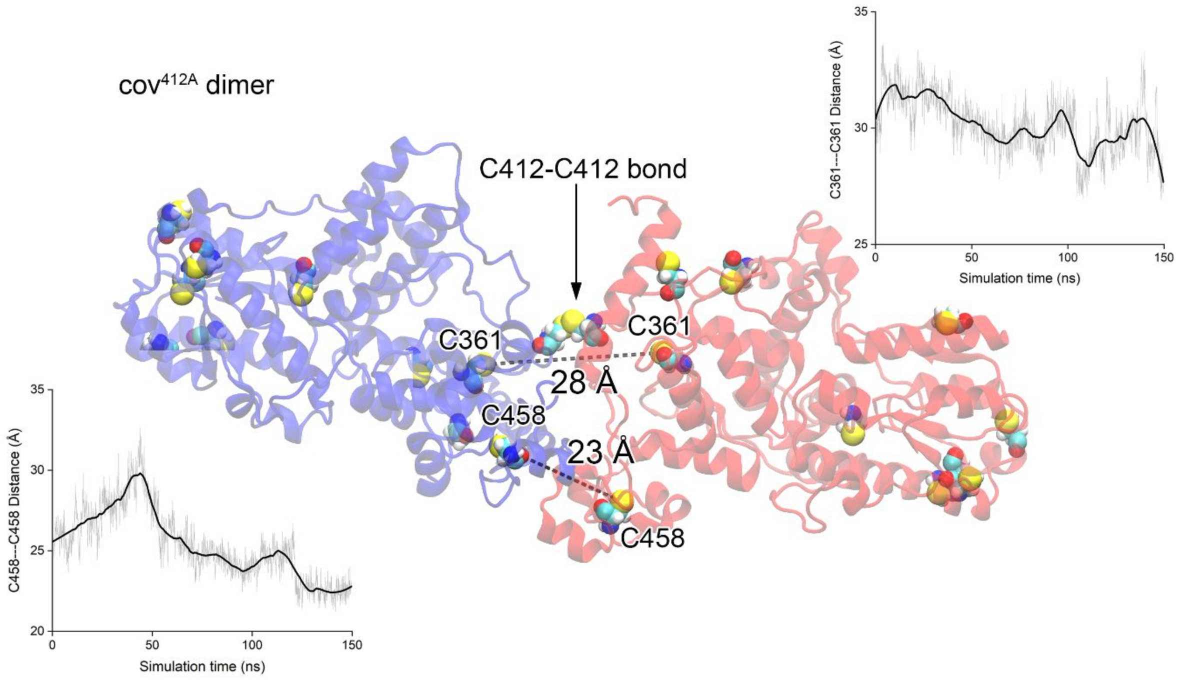

Figure S16: cov^{412A} dimer with the two monomers colored in red and blue for clarity. All of the cysteine residues are shown in VDW representation, and some distances are noted between the sulphur atoms at the end of the simulation. Insets show the distance plots between the C361-C361 and C458-C458 cysteines plotted against simulation time.

Distance analysis between cysteine residues

To determine which cysteine residues are in close contact, the six most surface-exposed cysteines (C317, C116, C189, C68, C412, C73) and C179 (see Table S3) were analysed in the 49 dimeric structures obtained using the docking tools. Tables S11-S17 summarise the distances between these cysteine residues. The tables are organised by the contact residue used in the docking procedure to produce the various families of structures. Distances between cysteines less than 10 Å are coloured green, and those between 10 Å and 20 Å are in yellow. Tables S18 and S19 summarise the distances between cysteines for complexes that were docked without defining a contact residue, in one case using ZDOCK[7,8], and in the other using M-ZDOCK[5] which was used for symmetric multimer docking. The names in the following tables (Complex *N*) are used internally only here and not in the rest of this paper.

Table S10: Analysis of inter-monomer distances between cysteine residues in *Er*Cry4a for the 10 complexes produced by ZDOCK, where Cys68 was used as a contact residue. The distance measures the separation of the sulphur atoms in the two cysteine residues.

**ZDOCK Contact residue Cys68**			
	Residue 1	Residue 2	Distance (Å)
Complex 1	361	361	31.93
	412	412	25.56
	313	313	44.3
	257	257	56.63
Complex 2	257	412	28.15
	179	458	44.66
	412	116	43.91
Complex 3	257	458	23.85
	257	458	23.85
	68	458	27.79
	257	412	29.03
Complex 4	361	412	37.72
	412	412	36.51
	257	257	41.62
Complex 5	412	412	35.96
	458	458	72.08
	257	257	43.01
Complex 6	412	412	14.23
	361	361	27.73
	317	317	38.97
	458	458	38.12
Complex 7	257	412	37.24
	361	313	38.98
	313	313	41.65
Complex 8	257	313	37.25
	68	412	36.98
Complex 9	313	313	17.1
	361	361	30.65
	317	317	35.04
	412	412	37.86
	116	116	36.98
Complex 10	116	458	41.95
	313	412	29.88
	317	361	36.43

Table S11: Analysis of inter-monomer distances between cysteine residues in *Er*Cry4a for the 10 complexes produced by ZDOCK, where Cys73 was used as a contact residue. The distance measures the separation of the sulphur atoms in the two cysteine residues.

**ZDOCK Contact residue Cys73**			
	Residue 1	Residue 2	Distance (Å)
Complex 1	317	412	37.77
	412	412	26.1
	361	361	30.52
Complex 2	412	257	29.53
	257	313	52.5
	361	116	45.41
	458	179	45.94
Complex 3	458	257	26.98
	257	458	27.13
	458	68	26.77
	257	412	30.63
Complex 4	313	458	46.37
	412	412	36.073
	361	412	36.023
Complex 5	412	361	39.61
	257	257	41.84
	361	361	42.1
Complex 6	412	412	15
	361	361	27.72
	458	458	38.13
	317	317	41.05
Complex 7	412	257	35.24
	313	313	40.77
	361	116	47.64
Complex 8	412	257	33.5
	412	179	34.87
	257	313	37.48
Complex 9	313	313	19
	317	317	33.78
	361	361	34.69
Complex 10	412	313	28.14
	361	313	28.78
	313	313	37.87

Table S12: Analysis of inter-monomer distances between cysteine residues in *Er*Cry4a for the 10 complexes produced by ZDOCK, where Cys116 was used as a contact residue. The distance measures the separation of the sulphur atoms in the two cysteine residues.

**ZDOCK Contact residue Cys116**			
	Residue 1	Residue 2	Distance (Å)
Complex 1	361	361	29.15
	412	412	27.25
	313	313	44.3
Complex 2	179	458	45.1
	257	412	28.45
Complex 3	458	257	24.66
	412	68	41.13
	257	457	26.61
	68	412	39.31
Complex 4	361	412	35.42
	458	313	46.65
	68	68	40.56
Complex 5	412	412	38.18
	412	361	41.23
	68	68	44.38
	257	257	43.01
Complex 6	412	412	17.2
	361	361	28.26
	317	313	37.88
	458	257	30.57
	317	317	38.56
Complex 7	257	412	34.83
	313	412	38.87
	313	313	38.78
Complex 8	257	313	37.48
	257	412	32.32
Complex 9	313	313	16.81
	361	361	30.56
	317	317	33.78
Complex 10	313	412	26.58
	313	316	29.44
	313	313	39.24
	116	458	40.52

Table S13: Analysis of inter-monomer distances between cysteine residues in *Er*Cry4a for the 10 complexes produced by ZDOCK, where Cys179 was used as a contact residue. The distance measures the separation of the sulphur atoms in the two cysteine residues.

ZDOCK Contact residue Cys179			
	Residue 1	Residue 2	Distance (Å)
Complex 1	412	412	26.06
	361	361	29.15
	313	313	45.8
Complex 2	412	257	29.83
	361	257	37.23
	458	179	45.1
	361	116	45.41
Complex 3	458	257	24.66
	257	458	24.77
	257	412	29.7
	412	257	31.31
Complex 4	257	257	42.02
	68	68	43.15
	361	361	45.79
Complex 5	412	412	36.98
	257	257	43.96
	361	412	42.6
Complex 6	412	412	17.18
	361	361	27.72
	458	257	34.18
	361	313	40.26
Complex 7	257	412	35.24
	313	313	39.28
	116	361	49.43
Complex 8	412	257	30.51
	361	179	38.14
	257	116	42.96
Complex 9	313	313	16.03
	317	317	35.6
	361	361	30.65
Complex 10	412	116	30.34
	361	313	29.83
	313	317	40.48

Table S14: Analysis of inter-monomer distances between cysteine residues in *Er*Cry4a for the 3 complexes produced by ZDOCK, where Cys189 was used as a contact residue. The distance measures the separation of the sulphur atoms in the two cysteine residues.

**ZDOCK Contact residue Cys189**			
	Residue 1	Residue 2	Distance (Å)
Complex 1	189	189	15.96
	68	189	20.11
	189	73	18.96
	189	68	18.14
	189	257	18.2
	116	458	32.46
Complex 2	189	189	15.88
	73	68	25.44
	68	73	25.58
	116	116	44.36
	116	257	35.81
Complex 3	189	458	8.6
	189	458	25.38
	257	257	28.55
	458	73	14.4

Table S15: Analysis of inter-monomer distances between cysteine residues in *Er*Cry4a for the 3 complexes produced by ZDOCK, where Cys317 was used as a contact residue. The distance measures the separation of the sulphur atoms in the two cysteine residues.

**ZDOCK Contact residue Cys317**			
	Residue 1	Residue 2	Distance (Å)
Complex 1	317	317	4.39
	313	313	20.03
	361	361	29.72
	317	313	14.66
	313	317	13.78
Complex 2	313	313	18.12
	317	317	21.93
	317	313	21.19
	313	317	21.68
	361	317	28.45
Complex 3	317	116	4.11
	458	313	16.01
	458	317	13.67
	412	313	24.08

Table S16: Analysis of inter-monomer distances between cysteine residues in *Er*Cry4a for the 3 complexes produced by ZDOCK, where Cys412 was used as a contact residue. The distance measures the separation of the sulphur atoms in the two cysteine residues.

**ZDOCK Contact residue Cys412**			
	Residue 1	Residue 2	Distance (Å)
Complex 1	412	412	31.74
	361	361	37.26
	313	313	40.47
Complex 2	458	458	17.72
	412	412	18.15
	361	421	21.83
	317	361	33.03
Complex 3	313	412	23.46
	361	361	23.01
	412	412	21.23
	412	317	23.9

Table S17: Analysis of inter-monomer distances between cysteine residues in *Er*Cry4a for the 10 complexes produced by ZDOCK, where no contact residue was specified. The distance measures the separation of the sulphur atoms in the two cysteine residues.

**ZDOCK NO Contact residue**			
	Residue 1	Residue 2	Distance (Å)
Complex 1	68	68	33.21
	257	257	40.03
	179	179	34.49
Complex 2	257	257	65.64
	412	412	55.1
	68	68	64.26
Complex 3	412	68	36.84
	458	68	37.34
	361	257	39.36
Complex 4	313	412	35.31
	257	257	41
	68	68	48.34
Complex 5	412	412	19.23
	361	68	15.65
	313	73	26.53
	317	189	29.74
Complex 6	257	257	43.35
	68	179	41.68
	179	68	39.77
Complex 7	257	412	39.18
	257	361	41.8
	361	313	39.63
Complex 8	458	317	22.01
	257	361	33.16
	68	412	28.62
	458	313	13.12
Complex 9	412	313	31.13
	361	313	32.41
	313	313	37.34
Complex 10	317	317	35.95
	313	361	29.48
	313	412	26.85
	313	313	33.94
	361	313	34.27

Table S18: Analysis of inter-monomer distances between cysteine residues in *Er*Cry4a for the 10 complexes produced by M-ZDOCK, where no contact residue was specified. The distance measures the separation of the sulphur atoms in the two cysteine residues.

**M-ZDOCK NO Contact residue**			
	Residue 1	Residue 2	Distance (Å)
Complex 1	189	189	61.87
	73	189	20.28
	189	73	20.35
Complex 2	313	116	21.87
	116	313	19.44
	116	317	19.69
	116	361	21.34
Complex 3	412	412	30.33
	361	361	31.3
	313	313	31.76
Complex 4	313	116	16.8
	317	116	20.71
	116	313	19.39
	116	317	19.7
Complex 5	313	116	19.74
	317	116	20.35
	116	313	19.74
	116	317	19.064
Complex 6	313	313	30.59
	257	361	42.41
Complex 7	189	189	30.59
	458	458	41.37
	458	458	30.92
	458	412	32.3
Complex 8	116	313	19.87
	116	317	20.45
	313	116	18.454
	116	361	20.915
Complex 9	116	317	5.09
	116	313	6.02
	313	116	8
	317	116	2.2
Complex 10	313	313	20.34
	116	313	21.54
	317	116	23.46

Molecular dynamics simulation protocol

Table S19: Summary of all the MD simulations performed. cov and ncov denote covalent and non-covalent dimers, respectively. aa stands for amino acid. cov^{D} is the dimer with two disulphide bonds (Cys116-Cys313 and Cys313-Cys116). ncov^{M} is the non-covalent dimer based on the structure of mouse Cry2.

Dimer	Statistical ensemble	Integration time step (fs)	Constrained atoms	Simulation time (ns)
**Initial equilibration**				
(cov317)^{A,B,3}	NPT	1	Protein except aa 310-320	1
	NPT	1	Backbone except 310-320	2
	NVT	1	None	2
(cov412)$^{1,2,3,A,B,6-8}$	NPT	0.1 or 1	Protein except aa 410-414	1
	NPT	1	Backbone except 410-414	15
	NVT	1	None	15
(cov189)	NPT	1	Protein	1
	NPT	1	Backbone	2
	NVT	1	None	3
cov(116^{A}313^{B}-313^{A}116^{B})= cov^{D}	NPT	1	Protein	1
	NPT	1	Backbone	5
	NVT	1	None	5
mouse-like = ncov^{M}	NPT	1	Protein	1
	NPT	1	Backbone	5
	NVT	1	None	5
ncov$^{1-3}$,ncov$^{A,5-9}$	NPT	2	None	2
**Production simulation**				
(cov317)$^{1-3}$/	NVT	2	None	400
(cov412)$^{1,2,3,A,B,6-8}$	NVT	2	None	150
(cov189)	NVT	2	None	100
cov^{D}	NVT	2	None	100
ncov^{M}	NVT	2	None	100

Involvement of the C-terminal in dimer formation

Table S20: Visualisation of computationally constructed dimeric *Er*Cry4a structures. Monomers are shown with different colours (green and grey), while partial C-terminal tails (residues 489-495) are shown in cyan.

Dimer name	Dimer visualisation	Involvement of CTT in dimerisation
cov^{317A}	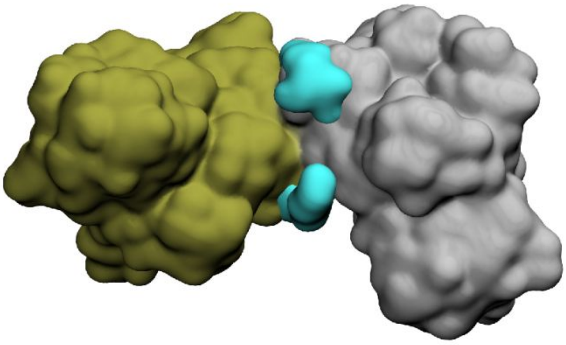	Minimally involved
cov^{317B}	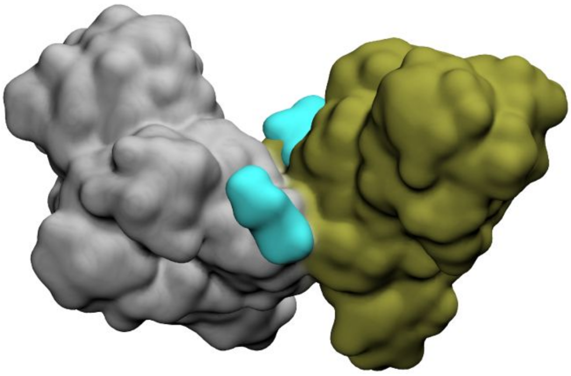	No
cov(317)^{3}	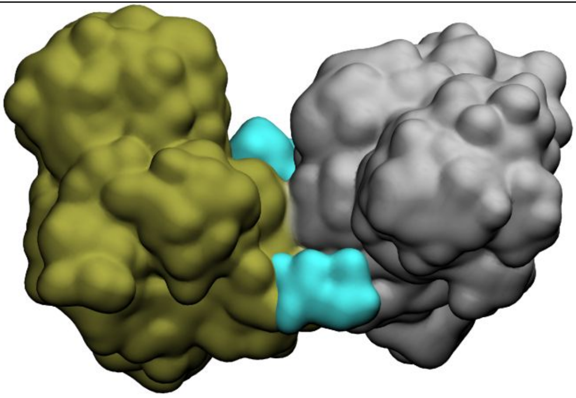	Yes

cov(412)^{1}	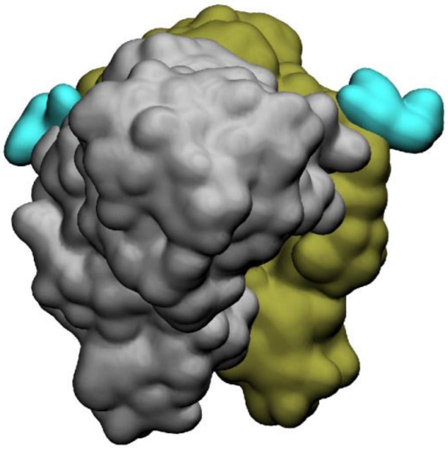	No
cov(412)^{2}	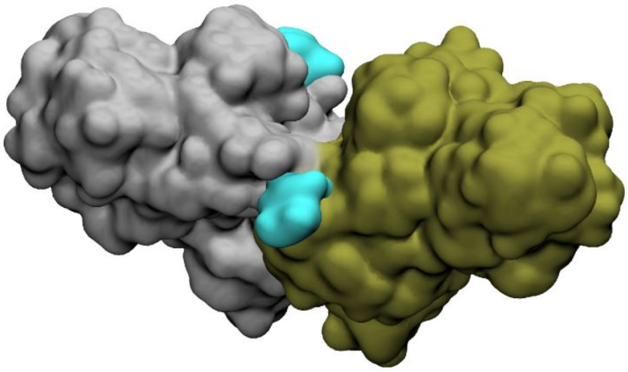	Minimally involved
cov(412)^{3}	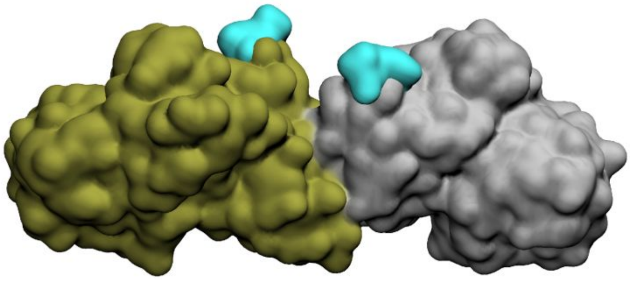	No
cov(412)^{4}= cov^{412A}	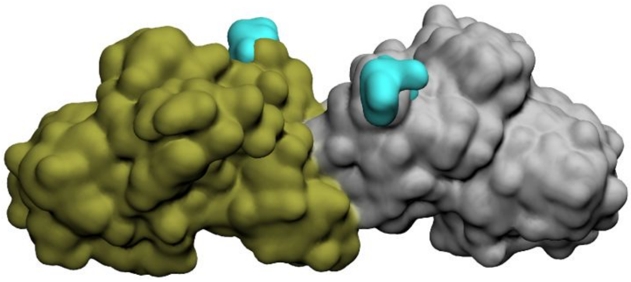	No

$cov(412)^5$= cov^{412B}	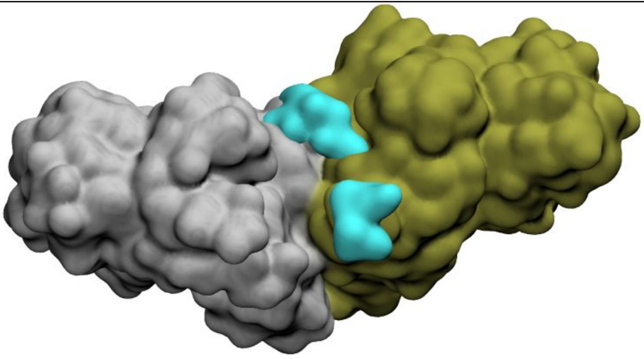	Minimally involved
$cov(412)^6$	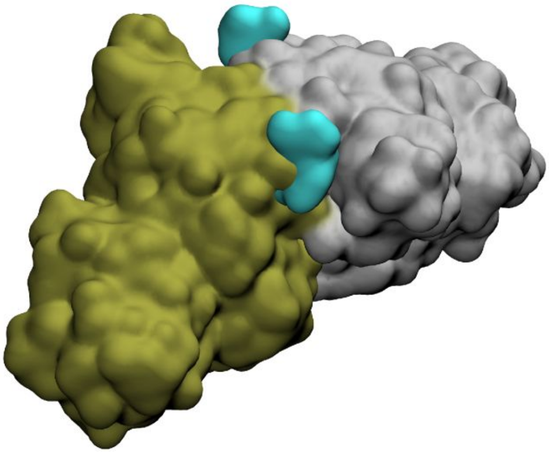	Minimally involved
$cov(412)^7$	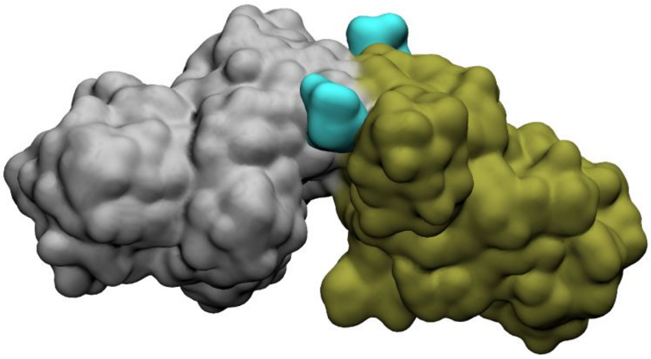	No
$cov(412)^8$	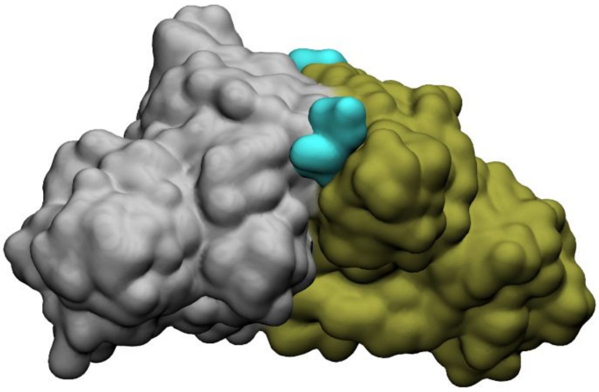	Yes

cov^{D}	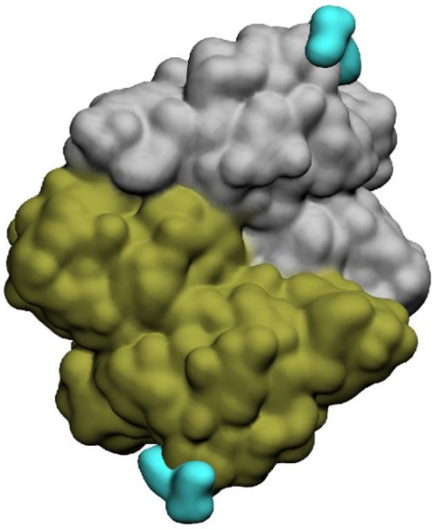	No
ncov^{M}	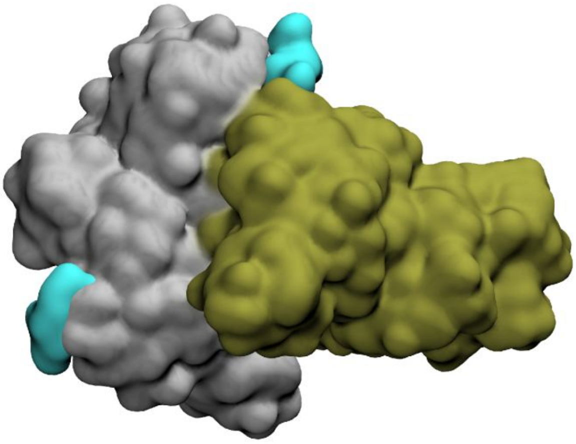	Minimally involved
cov^{189A}	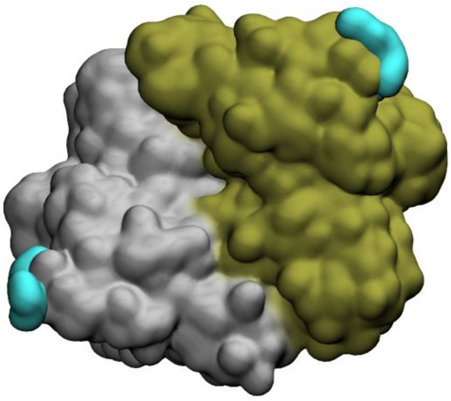	No

Comparison of cross-link experiment with MD simulation data

Table S21: Distances (Å) between Cα atoms of lysine residues averaged over the whole trajectory. Values that satisfy the criterion for the DSBU or DSSO linkers are shown in green.

**DSBU**	**cov^{412A}**		**ncov^{M}**	
	Min	Max	Min	Max
K152-K152	36.9	54.1	50.5	55.8
K234(cry1)-K429(cry2)	45.2	58.4	49.9	61.1
K234(cry2)-K429(cry1)	41.7	54.8	13.4	20.5
K234(cry1)-K429(cry1)	19.2	30.4	22.1	32.8
K234(cry2)-K429(cry2)	21.4	35.0	19.0	30.0

	**cov^{412B}**		**cov^{317A}**	
	Min	Max	Min	Max
K152-K152	50.4	59.1	51.9	62.3
K234(cry1)-K429(cry2)	46.7	51.9	55.3	68.8
K234(cry2)-K429(cry1)	55.0	67.8	55.2	72.8
K234(cry1)-K429(cry1)	19.5	33.4	18.7	33.7
K234(cry2)-K429(cry2)	26.8	33.5	16.9	35.9

	**cov^{D}**		**cov^{317B}**		**ncov^{A}**	
	Min	Max	Min	Max	Min	Max
K152-K152	35.2	37.5	53.1	62.9	43.3	52.6
K234(cry1)-K429(cry2)	80.5	87.7	67.5	85.9	10.5	25.3
K234(cry2)-K429(cry1)	83.0	87.5	66.2	74.2	53.3	63.4
K234(cry1)-K429(cry1)	20.7	25.8	18.7	30.9	26.5	40.7
K234(cry2)-K429(cry2)	17.0	35.4	18.6	28.2	27.7	37.9

**DSSO**	**ncov^{A}**	
	Min	Max
K234(cry1)-K519(cry2)	35.1	56.5
K234(cry2)-K519(cry1)	14.7	27.0
K234(cry1)-K519(cry1)	12.7	33.9
K234(cry2)-K519(cry2)	11.9	26.2

References

(1) Pepys, M. B.; Hirschfield, G. M.; Tennent, G. A.; Gallimore, J. R.; Kahan, M. C.; Bellotti, V.; Hawkins, P. N.; Myers, R. M.; Smith, M. D.; Polara, A.; Cobb, A. J. A.; Ley, S. V.; Aquilina, J. A.; Robinson, C. V.; Sharif, I.; Gray, G. A.; Sabin, C. A.; Jenvey, M. C.; Kolstoe, S. E.; Thompson, D.; Wood, S. P. Targeting C-Reactive Protein for the Treatment of Cardiovascular Disease. *Nature* **2006**, *440*, 1217–1221.

(2) Mädler, S.; Bich, C.; Touboul, D.; Zenobi, R. Chemical Cross-Linking with NHS Esters : A Systematic Study on Amino Acid Reactivities. **2009**, *44*, 694–706.

(3) Zoltowski, B. D., Chelliah, Y., Wickramaratne, A., Jarocha, L., Karki, N., Xu, W., Mouritsen, H., Hore, P. J., Hibbs, R. E., Green, et al. (2019). Chemical and structural analysis of a photoactive vertebrate cryptochrome from pigeon. *PNAS,* 116(39), 19449–19457.

(4) Shao, K.; Zhang, X.; Li, X.; Hao, Y.; Huang, X.; Ma, M.; Zhang, M.; Yu, F.; Liu, H.; Zhang, P. The Oligomeric Structures of Plant Cryptochromes. *Nat. Struct. Mol. Biol.* **2020**, *27*, 480–488.

(5) Palayam, M.; Ganapathy, J.; Guercio, A. M.; Tal, L.; Deck, S. L.; Shabek, N. Structural Insights into Photoactivation of Plant Cryptochrome-2. *Commun. Biol.* **2021**, *4*, 1–11.

(6) Pierce, B.; Tong, W.; Weng, Z. M-ZDOCK: A Grid-Based Approach for C_n Symmetric Multimer Docking. *Bioinformatics* **2005**, *21*, 1472–1478.

(7) Chen, R.; Li, L.; Weng, Z. ZDOCK : An Initial-Stage Protein-Docking Algorithm. *Proteins Struct. Funct. Genet.* **2003**, *87*, 80–87.

(8) Pierce, B. G.; Wiehe, K.; Hwang, H.; Kim, B. H.; Vreven, T.; Weng, Z. ZDOCK Server: Interactive Docking Prediction of Protein-Protein Complexes and Symmetric Multimers. *Bioinformatics* **2014**, *30*, 1771–1773.
